# Social science – STEM collaborations in agriculture, food and beyond: an STSFAN manifesto

**DOI:** 10.1007/s10460-023-10438-2

**Published:** 2023-05-01

**Authors:** Karly Burch, Julie Guthman, Mascha Gugganig, Kelly Bronson, Matt Comi, Katharine Legun, Charlotte Biltekoff, Garrett Broad, Samara Brock, Susanne Freidberg, Patrick Baur, Diana Mincyte

**Affiliations:** 1 University of Auckland, Auckland, Aotearoa New Zealand; 2 University of California, Santa Cruz, USA; 3 University of Munich, Munich, Germany; 4 University of Ottawa, Ottawa, Canada; 5 National Farm Medicine Center at Marshfield Clinic Research Institute, Marshfield, USA; 6 Wageningen University, Wageningen, Netherlands; 7 University of California, Davis, USA; 8 Rowan University, Glassboro, NJ USA; 9 Yale University, New Haven, USA; 10 Dartmouth College, Hanover, USA; 11 University of Rhode Island, Kingstown, USA; 12 City University of New York, New York City, USA

**Keywords:** Interdisciplinarity, Interdisciplinary and transdisciplinary research, Science and technology studies (STS), AgTech, FoodTech and agri-food tech, Technology research and development (R&D), Agri-food

## Abstract

Interdisciplinary research needs innovation. As an action-oriented intervention, this Manifesto begins from the authors’ experiences as social scientists working within interdisciplinary science and technology collaborations in agriculture and food. We draw from these experiences to: 1) explain what social scientists contribute to interdisciplinary agri-food tech collaborations; (2) describe barriers to substantive and meaningful collaboration; and (3) propose ways to overcome these barriers. We encourage funding bodies to develop mechanisms that ensure funded projects respect the integrity of social science expertise and incorporate its insights. We also call for the integration of social scientific questions and methods in interdisciplinary projects *from the outset*, and for a genuine curiosity on the part of STEM and social science researchers alike about the knowledge and skills each of us has to offer. We contend that cultivating such integration and curiosity within interdisciplinary collaborations will make them more enriching for all researchers involved, and more likely to generate socially beneficial outcomes.

## Introduction

Interdisciplinary research needs innovation. Many government funders of STEM-based research and technology development now expect or even require interdisciplinary or transdisciplinary approaches (e.g., European and Commission 2020). Funding calls often ask that research teams include social scientists within these collaborations, especially in the domain of food and agriculture technology where past innovations have proven controversial—such as genetic engineering (European Commission 2007; Wynne 2001). Yet despite this recognition of the value of social scientists’ expertise, inter- and transdisciplinary collaborations in agri-food research remain a challenge. Too often social scientific expertise is either treated as tangential to the core aims of science and technology research or disregarded entirely.

As an action-oriented intervention, this Manifesto begins from our experiences as social scientists working within inter- and transdisciplinary science and technology research collaborations in agriculture and food. We draw from these experiences to: 1) explain what social scientists contribute to interdisciplinary agri-food tech collaborations; (2) describe barriers to substantive and meaningful collaboration; and (3) propose ways to overcome these barriers.

The problems that food and agricultural research aim to address are as familiar as they are urgent: climate change adaptation, sustainable food production through digital farming tools, or most recently how to address crises such as supply chain disruptions and food supply shortages resulting from the COVID-19 pandemic and the war in Ukraine. Somewhat paradoxically, it is this very familiarity and urgency that make STEM – social science collaborations in the agri-food domain particularly challenging. This is because agri-food problems attract STEM researchers, entrepreneurs and funders who may feel confident that they understand a problem’s underlying cause and are compelled to deliver technological fixes as quick solutions (Belasco 2004; Fairbairn et al. 2022; Li 2007).

While funders and STEM scholars may bring moral commitments and technological skill to agri-food technology projects, food and agriculture remain an “intimate” issue for members of the public and for farmers: everyone eats and many people around the world are still involved in primary production (Winson 1994). Thus, despite the good intentions behind them, agri-food technologies have not always or universally been welcomed by relevant end-users, stakeholders, rights holders (e.g., Indigenous treaty partners) and members of the wider public. Most notably, while a success by some measures, many people critique the Green Revolution where attempts to bring high yielding varieties to many parts of the so-called “developing” world led to many negative social and environmental consequences—such as increased dependence on chemical inputs and a loss of livelihood for many farmers (Patel 2013). Thus, technological development in agri-food is a site rife with possible tension between technological goals and societal realities and needs.

The underlying tensions afflicting agri-food technological development illustrate why it is so critical to have social scientists involved in innovations *while they are being developed* rather than after the fact. Consider the fate of GMOs which are a specter looming over innovation today. Social scientists have shed nuanced light on how public resistance to GM was less about the technology itself and more about its social and environmental consequences: privatization of seeds, corporate consolidation, and untested environmental consequences ( Bronson 2018a; Schurman and Munro 2010). Had social scientists been able to engage with GMO developers to address these concerns during innovation processes, the outcome might have been different and innovation today would not have this troubled legacy. Indeed, many STEM scholars we engage with refer to the public resistance to GMOs as something they fear encountering within their research. STEM scholars want to develop technologies that will be accepted and adopted, but this will entail an early integration of the kinds of insights that social scientists bring—notably about public values, needs and concerns which are often non-obvious to technical experts. However, such STEM – social science collaborations are challenged by disciplinary differences which are often not explicitly recognized or discussed within interdisciplinary research teams: While some social scientists may work in ways more familiar to STEM researchers (agricultural economists or cooperative extension scientists in the case of agri-food), most academic social science is steeped in critical analysis that requires qualitative explanation and contribution to theoretical debates in the field.

Given the stakes of technology development and scientific research in the domain of agri-food, we want this Manifesto to be shared with both funding agencies and STEM project teams seeking to collaborate with social scientists in this field. As an interdisciplinary journal, *Agriculture and Human Values* offers an ideal intellectual space to spark such conversations. That said, we also believe these insights will be valuable for anyone funding or participating within interdisciplinary science and technology research collaborations. We hope that our experiences and recommendations can practicably act as guidelines for improving both researcher experiences within and the research outcomes of interdisciplinary collaborations.

We encourage funding bodies to develop mechanisms that ensure funded projects respect the integrity of social science expertise and incorporate its insights. We also call for the integration of social scientific questions and methods in interdisciplinary projects *from the outset*, and for a genuine curiosity on the part of STEM and social science researchers alike about the knowledge and skills each of us has to offer. We contend that cultivating such integration and curiosity within interdisciplinary collaborations will make them more enriching for all researchers involved, and more likely to generate socially beneficial outcomes.

The authors of this Manifesto come from several countries and disciplines, including Science and Technology Studies (STS), Food Studies, Sociology, Political Ecology, Geography, Communication Studies, Agroecology, History, Cultural Studies, Anthropology, and American Studies. We all belong to the Science and Technology Studies Food and Agriculture Network (STSFAN). While our research methods are largely qualitative, a number of us also have backgrounds in quantitative social science and natural science disciplines, as well as the critical humanities. We draw from our experiences participating in 40 inter- and transdisciplinary science and technology projects, grant proposals and collaborations in food and agriculture based out of the United States, Canada, Aotearoa New Zealand, Germany, and at the European Union level, as well as from many conversations with colleagues who have had similar experiences.

To begin, we describe what social scientists bring to science and technology collaborations. We then discuss barriers to successful collaborations, and suggest concrete ways to overcome or at least mitigate them. We end with a short reflection on how to ensure interdisciplinary research continues developing in ways that generate societal benefits.

## What do social scientists contribute to science and technology collaborations?

History has shown that while scientific and technological innovations in food and agriculture can generate socially beneficial outcomes (e.g., decreasing the need for agricultural workers to engage in physically unhealthy tasks), the failure to account for societal values and needs *early on* in the design of an innovation often leads to unanticipated social and environmental problems (e.g., consolidating corporate power at the expense of farmers and agricultural workers) (Clapp 2012; see also Stilgoe et al. 2013). Social scientists can help to prevent such problems through several types of contributions to science and technology research collaborations. These include:

- **Insights into the history of agri-food innovations and their effects.** This includes knowledge about how science and technology has affected different social groups: industry representatives, farmers, agricultural workers, and consumers, among others (e.g., Bronson 2015; Kloppenburg 2005).
- **Insights into current social, economic, cultural, environmental and political contexts that shape, and are shaped by, science and technology.** In the field of agri-food, social scientists have investigated the wider societal landscape that influences agricultural and food technologies to illuminate the unspoken cultural assumptions, socioeconomic relations, and ways of knowing that are often implicitly embedded in, and shaped by, science and technology projects (Bronson and Knezevic 2016; Burch and Legun 2021; Carolan 2018; Chiles et al. 2021; Gardezi and Arbuckle 2020; Gugganig 2017; Guthman 2019; Kenny and Regan 2021; Legun 2015; Legun and Burch 2021). Bringing those assumptions to light can allow explicit deliberation within interdisciplinary collaborations—that is, researchers’ own social locations and cultural beliefs that may inadvertently shape their research relations and outputs (Tuck and Yang 2014).
- **Methodological expertise in how to study the values and needs of relevant end-users and wider publics.** Such methods can help STEM scholars better understand the needs and values of the social groups that they intend their science and technology to benefit (Burch et al. 2022a; Lezaun and Soneryd 2007; Wynne 2006). Put simply, people sometimes engage with technologies in unexpected ways (see de Laet and Mol 2000). Social scientists’ methodologies can help STEM researchers to understand why, and to incorporate this understanding into their own work.
- **Strategies to promote meaningful forms of public engagement.** Social scientists can work with STEM scholars to design projects that incorporate public feedback—especially the negative kind—early on in the research process. This provides opportunities for STEM scholars to address societal concerns *before* wide public controversy ensues. Such research practices could include community-led innovation (Liboiron 2017, 2021), deliberative engagement in innovation (Wilsdon and Willis 2004), critical technology assessment (CTA) (Schot and Rip 1997; see also Barben et al. 2008), and responsible innovation (Bronson 2019; Guston et al. 2014; Mamidipudi and Frahm 2020; Owen et al. 2013; Stilgoe et al. 2013; von Schomberg 2011).
- **Suggestions on how to improve funding policies and research practices.** Social scientists have the expertise and skills to analyze barriers affecting research processes and can provide policy recommendations to improve funding decisions and the experiences of research collaborators and participants. For instance, they can point out barriers created by intellectual property protocols or other structural and cultural barriers that are preventing socially beneficial science and technology research (Bronson 2018a; Burch et al. 2022b; Carolan 2018; Glerup et al. 2017; Liboiron 2017). This could include suggestions on how technologies can be built in responsible and transparent ways, or collaboratively designed (co-designed) by communities and members of the public—instead of reflecting the values of only private profit-oriented actors.

## Barriers and suggestions for improving interdisciplinary collaborations and their outcomes

We have identified six structural barriers and misunderstandings stemming from our collective experience and suggest ways to address them. These suggestions are based on our recognition that while there are structural aspects that need to change about research funding (see Frickel et al. 2016), various steps can still be taken to mitigate some of these barriers within interdisciplinary collaborations.

### Barrier 1: social science is treated as an add-on to science and technology research

In our experience, inter- and transdisciplinary science and technology research collaborations often include social scientists only after research priorities have been set. This is too late. In such situations social scientists’ work becomes secondary to a project’s technical objectives, which consequently do not benefit from the kind of social scientific expertise described above. For example, some of us have been invited onto large inter- and transdisciplinary projects only weeks (or days!) before their grant submission deadlines. We received generic emails that were clearly aimed at recruiting social scientists regardless of their specific expertise. These sorts of ex post facto and tokenistic invitations to collaborate make it impossible for social scientists to contribute meaningfully.

#### Suggestion 1: build relationships with social scientists and base technology projects on preliminary social scientific studies

Improving how social scientists are recruited within science and technology collaborations is an important first step to preventing the last-minute, tokenistic inclusion of social scientists who may not have the expertise necessary to support a particular research project. The first way to cultivate genuine interdisciplinary collaborations is for STEM project leaders to build relationships with the appropriate social scientists—those whose expertise aligns with the proposed project—long before funding deadlines. But the timing of relationship-building is not the only thing that needs to change. We urge funders to require that research proposals for technology development include empirical evidence of diverse societal needs—as opposed to the stated needs of powerful groups, such as industry partners. This means that funders should be willing to fund preliminary studies on the needs of the different stakeholders and rights holders (e.g., Indigenous treaty partners) who might benefit from or be affected by any proposed technologies (Bronson 2018b; Burch et al. 2022a).

These preliminary studies also open-up opportunities for stakeholders and rights holders to propose technologies that would be most valuable to them, so that science and technology projects can serve the needs of particular communities—instead of expecting communities to adopt the ideas of researchers or other powerful actors (Liboiron 2021). The studies must also consider whether a proposed technology is necessary in the first place. In other words, just because a given technology *can* be made does not necessarily mean that it *should* (de Saille and Medvecky 2016). As an accountability mechanism, funding institutions should consider a feedback section on research proposals from relevant stakeholders, rights holders and social scientists to ensure adequate evidence is provided for the appropriateness and necessity of the proposed technology.

### Barrier 2: social scientists are expected to adopt positivist frameworks

Positivism, or logical empiricism, is a scientific philosophy that emerged in the mid-nineteenth century and still dominates most STEM fields (Harding 2015). In brief, it prioritizes quantitative measures of impact, which often involve hypothesis-testing aimed at achieving a desired outcome (e.g., how to develop a new seed variety or robotic technology). By contrast, many social scientists draw from epistemological frameworks such as phenomenology, critical realism, material semiotics and actor network approaches. These qualitative frameworks draw attention to the political and economic structures, social relationships, and cultural meanings and values that positivist approaches cannot easily capture, and that influence how people experience and respond to scientific and technological developments. In many ways social scientists are interested in the very questions left out of positivist frameworks, such as what social and cultural values might influence which technologies are funded, diverse feelings and experiences associated with new technologies, or people’s reception to a particular technology.

In our experience, we have been confronted with the dominance of positivist framings and quantitatively measurable impacts which has manifested in being asked questions such as, “how does your research support our research aims?” or “how can you help society understand the benefits of *x* technology?” Such framings supply pre-determined research outcomes and orientations (pro-technology) as opposed to problematics that can be contextualized, explored and explained (core aspects of social scientific scholarship). In other cases, co-authors have been explicitly told that their research is only useful if it helps industry partners to get a technology to market with promises of wide adoption. Some social scientists are explicitly asked to stick to “objective” data points, even when engaging with highly value-laden techno-scientific issues. In other words, social scientists are often expected to sideline their own knowledge frameworks and disciplinary commitments. This, in effect, decreases opportunities for STEM scholars to remain responsive to the needs of relevant societal actors, or to integrate valuable critical insights into their research processes.

#### Suggestion 2: integrate social scientific leadership early in project planning and foster respectful approaches to bridge disciplinary differences

The design of a truly interdisciplinary project ought to recognize that all participating disciplines bring something valuable to the project—and that one discipline is not subservient to the others (York 2018). It also should recognize that project members are not only expected to contribute to the project but also to their own fields and the communities they engage with—as this is where their research will be evaluated. As mentioned, most academic social science is steeped in critical analysis that requires qualitative explanation and contribution to relevant theoretical debates. Trust and respect for these disciplinary differences in research philosophy, epistemology, and methodology must therefore be cultivated at a research collaboration’s outset. Doing this will require the fostering of mutual trust among technical and non-technical collaborators.

It is essential that project leaders from STEM and social science disciplines have open and honest discussions about what each field will bring and will need, and to ensure those contributions and requirements are built into research protocols. Funding institutions could require these discussions be explicitly documented within funding applications. At the same time, integrating social scientific leadership early in project planning could enable a stronger entry point for both social scientists and STEM scholars. This is because a better understanding of social dynamics and contexts means the most relevant research questions (whether technical or social) can be asked and answered within the research process.

### Barrier 3: STEM research priorities preclude the free expression of social scientists’ ideas and critiques

Social scientists and STEM scholars often define project success differently. For many social scientists, success is more about how well a research project accounted for a diverse set of values, needs and concerns. For many STEM researchers, a project succeeds when it delivers a proof of concept. Too often, this latter definition of success prevents social scientists from sharing their own findings within interdisciplinary collaborations. Many of us have learned the hard way about the possibility of reprisals and censorship.

In one case, when a social scientist openly shared in a presentation that she felt like she was being included in her interdisciplinary collaboration in a tokenistic way, the project lead abruptly ended her talk instead of allowing her to express what she could offer. In another case, a social scientist shared interview findings internally that questioned the approach of the project, received passive mixed responses from the team, and then published findings openly to project partners. The project lead subsequently reduced her fieldwork support and demanded that all future writing be vetted before publication. Indeed many of us have encountered the requirement that our publications be pre-approved by STEM colleagues, often as part of intellectual property agreements which aim to prevent unintentional invention disclosures. Unfortunately, there are additional negative outcomes for the career of a social scientist who is implicitly encouraged to self-censor: such censorship hinders their ability to fulfill contract obligations and to publish their work in high-ranking social science journals—where rigorous social analysis is an expectation.

#### Suggestion 3: make critical discussions a regular part of research processes

If research collaborations are to produce societal benefits, they need to tolerate and indeed welcome social scientific critique. Project leadership can begin by establishing mechanisms to handle conflicts and support constructive dialogue among disciplinary teams. Such mechanisms might include a set of shared principles or values for how the team wants to work together, or activities that build relationships robust enough to withstand the discomfort of critique (Burch et al. Forthcoming). They could also include workshops that facilitate productive conflict for the purpose of building mutual understanding, boundaries and respect among team members. We recommend that research collaborations develop these mechanisms at the outset of a project, and review them regularly with input from all team members. Funding institutions should encourage the establishment of such protocols, as well as reflections on their outcomes in annual project reports.

### Barrier 4: “willingness to adopt” and “public acceptance of technology” are considered the most relevant social scientific research questions when it comes to technology development

Social scientists can offer many insights into the social structures, relationships and values that affect the origin, design, implementation and adoption of new food and agricultural technologies. Yet projects developing these technologies often bring social scientists on board only to address questions about the final stage: i.e., *What informs a grower’s willingness to adopt x technology? What will improve the public acceptance of x technology?* Members of our writing team have participated in a wide variety of technology research and development projects, and very rarely have we been invited to engage in questions related to technology design. While research into the cultural, social, political, and economic contexts of adoption and acceptance has value (e.g., Broad et al. 2022; Comi 2019; 2020; Higgins et al. 2017; Legun and Burch 2021), so does social scientific research on other aspects of technology development (Fielke et al. 2022). Projects that narrowly define the scope of social science questions simultaneously limit social scientists’ career advancement and fail to leverage the full suite of expertise and skills available within an interdisciplinary collaboration.

#### Suggestion 4: provide social scientists with independent budgets and the autonomy to design their own research questions

For many interdisciplinary collaborations, the proposal and funding process operates as a de facto contract among team members and a roadmap for the scientific value and wider impact of our research. Just as with other aspects of the scientific endeavor, social scientists should have significant input, if not final say, over the budget items associated with their data collection and analyses to ensure funding is put to adequate use and in appropriate timelines.

Further, as experts of their own discipline, social scientists should be allowed to identify and articulate research questions relevant to the project that are not limited to questions of adoption and diffusion, such as:

- What technologies would be most valuable in addressing the needs of x community?
- What existing knowledge do diverse publics have about the nature, benefits, and drawbacks of x technology, as well as the broader context in which it is being developed?
- What underlying cultural assumptions inform x technology’s development and implementation?
- What power relations are likely to emerge or be entrenched through the adoption of x technology?
- What would make x technology socially and environmentally sustainable?
- How is intellectual property being managed within a research collaboration, and how might this affect technology design, adoption and use?
- How is data being managed within technology design and use? How might it be done differently to promote more equitable outcomes?

Suggesting that social scientists have autonomy in developing research questions and agendas is not tantamount to leaving them to their own devices. Social scientific expertise and findings need to be integrated into the project so that relevant social questions will be addressed while there is still an opportunity to shape science and technology processes. All research collaborators must actively engage in this integration process, as social scientists cannot and should not be responsible for generating socially beneficial outcomes on their own (Viseu 2015).

### Barrier 5: an expectation that social scientists represent “society” within a project

The idea that social scientists represent “the public” or “society” in science and technology research collaborations (Lezaun and Soneryd 2007; Wynne 2006) often leads to the neglect of relevant end-users, stakeholders and rights holders in technology design processes. As STS scholars have demonstrated, sometimes technology designers unwittingly project particular fears, ideas, or values onto the public that may not exist (Irwin 2001). Treating social scientists as proxy publics forecloses an opportunity for STEM researchers to examine their own assumptions about “publics” or “society,” and to gather empirical evidence on how particular groups frame a respective issue (Marres 2007).

As an example, one of the members of our writing team asked her project lead, a STEM researcher, about opportunities to interview possible end-users about whether the technology under development could adequately address their needs. The project lead said that such research was beyond the scope of the project and that she would need to apply for additional funding. This type of response is not uncommon, and it implies a disinterest in a technology’s end-users and society more broadly. Such disinterest often backfires on research teams by leading to the public rejection of a technology, or the generation of social and environmental problems which, because they were not adequately investigated, cannot be classified as “unintended consequences” (Parvin and Pollock 2020).

#### Suggestion 5: plan projects so they are able to integrate social scientific findings on public values and needs into technology design processes

If projects are concerned with public uptake of technologies in development, there must be funding mechanisms and incentives for assessing public uptake. Social scientists have an essential role to play in researching the values and needs of social groups relevant to science and technology research, as opposed to acting as proxies for such essential empirical insights. We thus propose that funders transform the impact-focused funding requirements currently associated with many transdisciplinary programs by requiring that: (1) social scientific research be part of all science and technology funding calls; (2) social scientists participate in writing science and technology funding calls; (3) social scientists serve on grant review committees; and (4) the values and needs of relevant social groups are investigated before, and during, proposed research projects. Funders should further develop accountability mechanisms for ensuring that social science findings on publics is heard, deliberated and at least nominally addressed within science and technology projects.

### Barrier 6: an expectation that social scientists must educate the “public” on technologies or translate scientific knowledge to “society”

When social scientists are not called upon to approximate publics within research processes, they are often expected to “educate” the public on the scientific and/or technological outputs of a research collaboration. This expectation assumes that social scientists are more skilled at dealing with the public than STEM researchers. It also assumes that researchers’ communication with the public will focus on making the public *accept* what scientists have said as opposed to deliberating the merits of a particular technology or scientific finding (either in public forums or with social scientists who have interviewed members of the public) (Cooke et al. 2017; Scheufele 2014; Seethaler et al. 2019). This latter assumption often relies on a view on public resistance now widely discredited in STS and related social science fields–namely that any public resistance reflects a “knowledge deficit” (Bronson 2018a; Bubela et al. 2009; Irwin and Wynne 1996).

As an example, when one of our co-authors collaborated with a high-tech vertical farm start-up for a public engagement event at a German museum, a local TV broadcast channel interviewed her (the social scientist) rather than the start-up representative to explain the technical system of indoor controlled farming (Waller and Gugganig 2021). While it is likely that the journalist chose the German speaker (the social scientist) over the English-speaking start-up representative, it epitomized a common confusion in transdisciplinary collaborations in science and technology, where social scientists not only turn into translators but representatives of a highly technical system (Lezaun et al. 2016). In this situation, she was expected to act as spokesperson for this high-tech food growth system—that is, eradicating a public knowledge deficit by educating the public about its positive aspects. This effectively sidelined her own research interest in how start-up representatives convey their technology in such a public setting, and the implications of that strategy. As a result, the project missed out on insights that could have been gained from the social scientist or through a more generative form of public deliberation.

#### Suggestion 6: plan for critical engagement with members of relevant social groups

In essence, understanding public concerns is not tantamount to eradicating them. To the contrary, the value that social scientists bring is to shed light on the potential disjunctures between how scientists imagine public concerns and what those concerns actually are—i.e., to illuminate what STS scholars have referred to as “misunderstood misunderstandings” (Goodin and Dryzek 2006; Irwin and Wynne 1996; Marres 2007). Ideally, research relationships with societal groups will be established before a project commences, through preliminary social scientific research (Suggestion 1) or by including relevant stakeholders and rights holders in collaborative design processes. Funders can request that researchers discuss how a proposed project will engage with relevant social groups and integrate their feedback into science and technology processes within their funding applications, and to report on what they have learned through these interactions.

## Conclusion

Social scientists have much to offer science and technology research in many sectors, but particularly in agriculture and food which are central to human existence while also having profound effects on the nonhuman world—what is commonly called “the environment.” It is precisely these stakes that make the agri-food domain prone to intense controversy.

We have written this Manifesto to shed light on the expertise and skills social scientists can offer interdisciplinary collaborations, but which may not be immediately apparent to funders and STEM scholars. At the same time, this Manifesto identifies barriers to meaningful interdisciplinary collaboration, which largely stem from social scientists being included only as tokenistic add-ons to science and technology projects, as well as from misunderstandings about what social scientists bring to the table. We suggest several ways in which these issues can be addressed both structurally (through changes in funding requirements) and within projects (through the actions of project leadership and collaborators). We see these suggestions as a useful starting point for ensuring: (1) interdisciplinary collaborations are meaningful and career-advancing for all researchers involved; and (2) projects generate socially beneficial outcomes.

We are eager to see metrics for project success that expand beyond the achievement of technical aspects or vague definitions of research impact. These new metrics should include inquiries into how well a diverse array of values, needs and concerns were accounted for within an interdisciplinary collaboration and embedded within the technologies being designed. We look forward to seeing how others engage with, and improve upon, our suggestions.
